# Supplementary material for: All-optical control and super-resolution imaging of quantum emitters in layered materials
Source: Nat Commun. 2018 Feb 28;9:874. doi: 10.1038/s41467-018-03290-0 (PMC5830405; doi:10.1038/s41467-018-03290-0)
Supplement: Supplementary file 1 — Supplementary Information [file 41467_2018_3290_MOESM1_ESM.pdf]

Supplementary information: All-optical control and super-resolution imaging of quantum emitters in layered materials

Mehran Kianinia, [#,†] Carlo Bradac, [#,†,*] Bernd Sontheimer,[‡] Fan Wang,[†] Toan Trong Tran,[†] Minh Nguyen,[†] Sejeong Kim,[†] Zai-Quan Xu,[†] Dayong Jin,[†] Andreas W. Schell,[§] Charlene J. Lobo,[†] Igor Aharonovich[†,*] and Milos Toth.[†,*]

† School of Mathematical and Physical Sciences, University of Technology Sydney, Ultimo, NSW, 2007, Australia.

‡ Institut für Physik, Humboldt-Universität zu Berlin, 12489 Berlin, Germany.

§ Department of Electronic Science and Engineering, Kyoto University, 615-8510 Kyoto, Japan.

* contributed equally

# correspondence to Carlo.Bradac@uts.edu.au, igor.aharonovich@uts.edu.au milos.toth@uts.edu.au

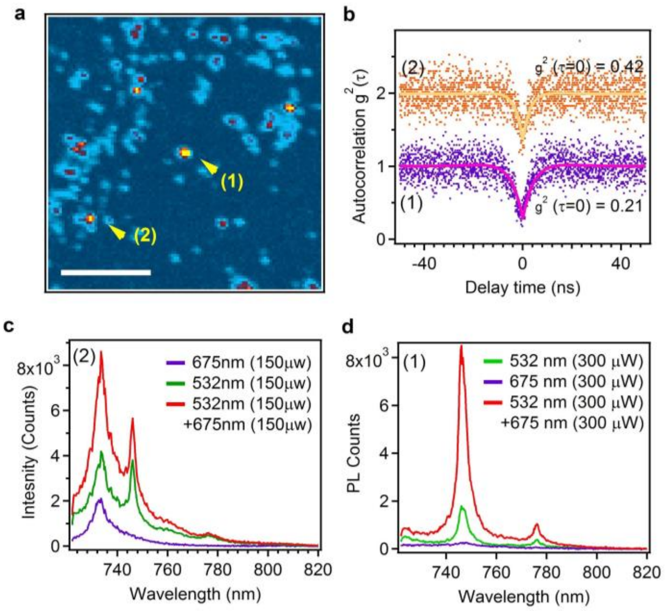

Supplementary Figure 1. Quantum emitters in hBN showing nonlinear behaviour. a) confocal scan of 30 x 30 µm^2 area of the sample revealing two quantum emitters. Scale bar is 10 µm. **b)** Autocorrelation measurements from emitter (1) and (2) with values of $g^2(\tau=0)$ of 0.21 and 0.42, respectively. The data is not background-corrected. **c, d)** PL spectra of emitters (1) and (2) under various power- and wavelength-dependent excitations, highlighting the nonlinear behaviour of the emitters (see main text).

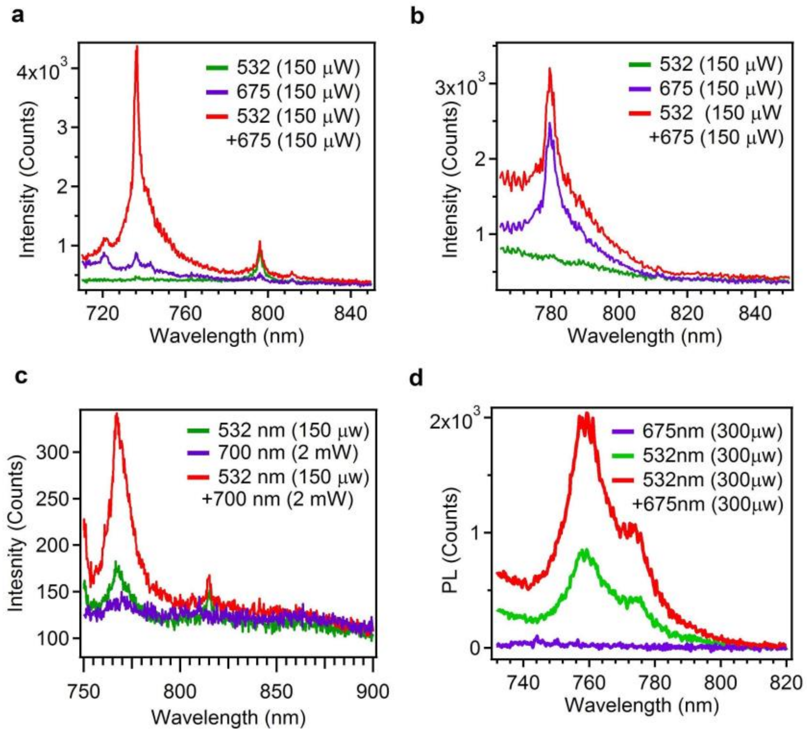

Supplementary Figure 2. Quantum emitters in hBN showing nonlinear behaviour. a–d) PL spectra of four different emitters in hBN under various excitation conditions. Note that in this case the emission is from ensembles of emitters.

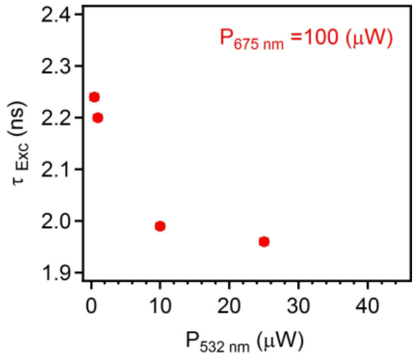

Supplementary Figure 3. Bunching values. Bunching time values extracted from the fitting to the autocorrelation data in Fig. 2a.

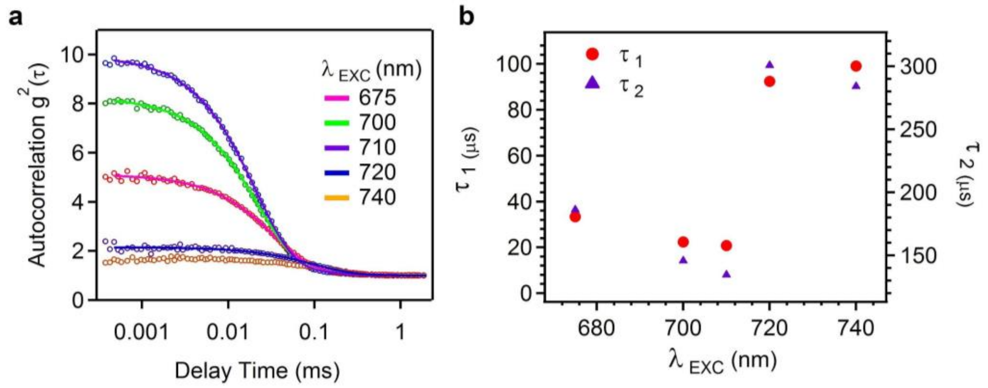

Supplementary Figure 4. Dependence of the metastable states time constants (τ_1 and τ_2) on the excitation wavelength. a) Autocorrelation measurement on long delay times, up to 1 ms, under various excitations from 675 to 740 nm. Solid lines are the fit with the four-level model. **b)** Values of τ_1 and τ_2 extracted from the fit to data in (a).

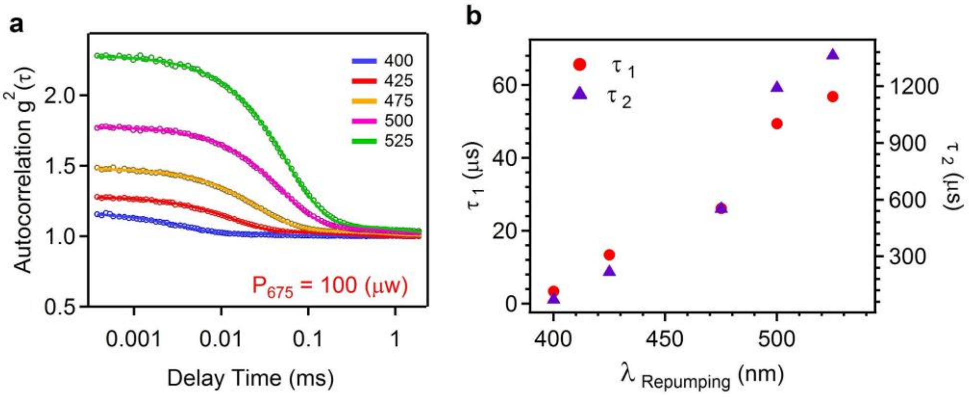

Supplementary Figure 5. Dependence of the intermediate/metastable states time constants (τ_1 and τ_2) with the wavelength of the repumping laser. a) Autocorrelation measurement on long delay times, up to 1 ms, under various repumping laser wavelengths, in the range 400–532 nm. The emitter was excited with 100 µW of the 675-nm laser in all measurements. Solid lines are fits to a four-level model. **b)** Change in τ_1 and τ_2 extracted from the fit to the data in (a).

Supplementary note 1: Emitters in hBN with nonlinear behaviour

In this work we surveyed several hBN emitters. The nonlinear behaviour presented in the manuscript was observed in a number of cases, including both single emitters (Supplementary Figure 1) and ensembles (Supplementary Figure 2). Only emitters with a zero phonon line (ZPL) above 700 nm were investigated (due to experimental constraints). Supplementary Figure 1a shows a typical confocal scan of the sample. The autocorrelation measurements from emitters (1) [violet] and (2) [orange] are shown in Supplementary Figure 1b. At zero delay time, $g^2(\tau=0)$ = 0.21 and 0.42 for (1) and (2), respectively, indicating that the emission is from single defects (the datasets in Supplementary Figure 1b are not background-corrected).

Both emitters showed the nonlinear behaviour presented in the manuscript under co-excitation with a 675-nm (or 708-nm) and a 532-nm laser, as shown in Supplementary Figure 1c and 1d (analogous to Fig. 1d of the main manuscript). In both spectra, the emission intensity under co-excitation with the 532-nm and the 675-nm laser [red trace] is (nonlinearly) higher than the emission intensity obtained via independent excitation with the 675-nm [violet trace] or the 532-nm [green trace] lasers. The same nonlinear behaviour was also observed for other emitters which did not show photon antibunching in the autocorrelation measurements, $g^2(\tau=0)$, hence referring to ensembles. Supplementary Figure 2 shows the PL spectrum of four different emitters in hBN under various excitation conditions. Note that while the autocorrelation measurements for single emitters support our hypothesis of the repumping mechanism discussed in the main text (which leads to the observed nonlinear behaviour), the same claim could not – in principle – be made in the case of ensembles where other effects might be at play (e.g. difference in absorption cross sections of the emitters).

Supplementary Note 2: Intermediate/Metastable states

The best fit to the autocorrelation data in Fig. 2a was achieved assuming the dynamic of a four-level system. The bunching time (τ_{Exc}) corresponding to the excited state lifetime is shown in Supplementary Figure 3.

To determine the relative dependence of the intermediate/metastable states lifetimes (τ_1 and τ_2) on the excitation wavelength, two additional experiments were performed. First, the second-order autocorrelation function was measured using excitation from a single laser at a fixed power (300 µW - Tisaph, M squared), and with wavelength ranging from 675 nm to 740 nm (Supplementary Figure 4). The data was fitted considering a four-level structure including two intermediate/metastable states; the value for the corresponding τ_1 and τ_2 were extracted accordingly. Notably, for excitations at 710 nm and below, the population of the metastable states is relatively high.

Next, autocorrelation measurements were performed by varying the wavelength of the repumping laser (NKT photonics, Fianium WhiteLase supercontinuum laser), while continuous excitation with the 675-nm laser was fixed (Supplementary Figure 5). For all measurements the power of the 675-nm laser was 100 µW and the power of the repumping laser was kept as 10 µW. Under these conditions, the transition rate to the intermediate/metastable states increased as the wavelength of the repumping laser increased. The data, again, fits well with a four-level system.
